# Self-compassion and English learning anxiety among college students: a moderated chain mediation model

**DOI:** 10.3389/fpsyg.2025.1697291

**Published:** 2026-01-16

**Authors:** Lingyan Zhou, Xiaojing Zhang, Jingyu Qiang, Cheng Xu

**Affiliations:** 1 School of Foreign Languages, Guangdong University of Petrochemical Technology, Maoming, Guangdong, China; 2 School of Psychology and Cognitive Science, East China Normal University, Shanghai, China

**Keywords:** English learning anxiety, experiential avoidance, gender differences, personal growth initiative, self-compassion

## Abstract

**Objective:**

English learning anxiety has become a significant factor affecting students' learning efficiency and academic performance, particularly among Chinese university students. As a psychological intervention, self-compassion has been shown to effectively alleviate anxiety. However, there is a lack of systematic empirical research on how self-compassion influences English learning anxiety through internal psychological mechanisms, especially in the context of Chinese culture. This study aims to explore the relationship between self-compassion and English learning anxiety among Chinese university students, focusing on the role of personal growth initiative and experiential avoidance as mediators, and examining gender differences as a moderator.

**Methods:**

This study used a questionnaire survey method to recruit 1,287 university students from China. Data were collected using the Self-Compassion Scale, Personal Growth Initiative Scale, Experiential Avoidance Scale, and English Learning Anxiety Scale. SPSS Process macro was used to analyze the relationships between self-compassion, personal growth initiative, experiential avoidance, and English learning anxiety, and to test the moderating effect of gender on these relationships.

**Results:**

The results indicated that self-compassion significantly negatively predicted English learning anxiety, and this relationship was mediated by personal growth initiative and experiential avoidance. Additionally, gender played a significant moderating role in the relationship between self-compassion and English learning anxiety, with female students being more likely to exacerbate anxiety through experiential avoidance when faced with academic pressure.

**Conclusion:**

This study provides theoretical support for interventions aimed at reducing English learning anxiety, particularly in the Chinese educational context where students generally experience high academic pressure. The findings suggest that educators should introduce self-compassion interventions in their teaching to help students regulate emotions, boost self-confidence, and reduce learning anxiety. Moreover, the presence of gender differences indicates that teachers should design personalized interventions based on students' gender characteristics.

## Introduction

Foreign language learning anxiety was first introduced by Horwitz in 1986, defined as the tension, nervousness, and worry experienced by learners in the process of foreign language acquisition, often accompanied by physiological responses of autonomic nervous system activation, such as sweating and palpitations (Horwitz et al., 1986). This type of anxiety is generally composed of three components: communication apprehension, test anxiety, and fear of negative evaluation. In the context of accelerating globalization and increasingly frequent international exchanges, English, as a global lingua franca, is not only essential for individual academic development and professional competitiveness but also constitutes a crucial component of the national education system. In China, English instruction has been emphasized since basic education, and college English is designated as a compulsory course in higher education. However, within a dual environment characterized by both “exam-oriented” and “instrumental” learning, students experience substantial academic pressure, and English learning anxiety has become increasingly salient. Existing studies have confirmed that foreign language learning anxiety is a prevalent psychological phenomenon that significantly undermines students' efficiency and outcomes in language learning. Among Chinese university students, this problem is particularly pronounced. Research has demonstrated that they frequently encounter challenges such as lack of linguistic environment, insufficient self-efficacy, and heavy classroom demands, all of which maintain anxiety at a persistently high level (Zeng et al., 2020). The negative impact of English learning anxiety manifests in multiple dimensions. For instance, students with higher levels of anxiety often show lower academic achievement (Liu, 2023; Said and Weda, 2018), perform more poorly in output-related skills such as speaking and writing (Özdemir and Seçkin, 2025), and exhibit reduced motivation and self-confidence, further hindering the development of their language proficiency and academic performance (Sukjairungwattana et al., 2025). Therefore, investigating the underlying causes of English learning anxiety and identifying effective intervention mechanisms are not only vital for optimizing English teaching practices and improving students' learning experiences and academic outcomes but also contribute to enhancing the overall quality of English instruction in higher education.

Prior research has highlighted the role of self-compassion in language learning contexts (Sönmez and Kurtoglu, 2021). For example, previous research has shown that students with different levels of self-compassion exhibit varying levels of anxiety, and that improving self-compassion can help reduce anxiety and enhance students‘ mental health (Deniz and Sumer, 2010; Kirkpatrick, 2005; Neff K. D., 2003). The research also found that certain methods can simultaneously improve learners' self-compassion, enhance their foreign language learning performance, reduce anxiety, and improve their mental health (Liu et al., 2025; Namaziandost et al., 2025). However, most existing studies have overlooked the internal mechanisms through which self-compassion affects English learning anxiety. Particularly in the highly intertwined cognitive-emotional context of language learning, there is no empirical research on how self-compassion can stimulate intrinsic growth motivation through cognition, reduce maladaptive emotion regulation, and thus alleviate English learning anxiety. Furthermore, some literature has found a relationship between foreign language learning anxiety and gender, with female gender being significantly associated with higher levels of foreign language learning anxiety, and women reporting significantly higher anxiety than men (Park and French, 2013; Stephenson, 2007). Yet in the Chinese sociocultural context, gender roles are shaped by both traditional norms and social expectations, leading to more pronounced gender differences in stress response, emotional management, and learning attitudes (Matud, 2004). Accordingly, this study also examines the moderating role of gender, aiming not only to fill the gap in gender research within the domain of language learning but also to reveal culturally grounded psychological mechanisms in the Chinese context.

Self-compassion, first proposed by Kristin Neff, refers to treating oneself with kindness, understanding, and care when encountering difficulties or setbacks, rather than engaging in harsh self-criticism. Neff conceptualized self-compassion as comprising three interrelated dimensions: self-kindness, common humanity, and mindfulness (Neff K., 2003). As self-compassion has been increasingly confirmed by research to have positive effects on individuals' academic performance and mental health, this concept has gained widespread attention in the field of educational psychology in recent years (Liu et al., 2025; Namaziandost et al., 2025; Wiliyanto et al., 2024). English learning anxiety, as a specific manifestation of foreign language learning anxiety described by Horwitz and colleagues, represents a complex emotional response elicited in classroom contexts (Horwitz et al., 1986). It typically involves self-cognition, beliefs, emotions, and behaviors, and is primarily composed of communication apprehension, test anxiety, and fear of negative evaluation (Horwitz et al., 1986). At its core, English learning anxiety is a psychological state closely associated with fear and anxiety. Empirical studies have shown that self-compassion is negatively correlated with anxiety, suggesting that it serves as a psychological buffer against anxious emotions (Hughes et al., 2021; Luo et al., 2023). Moreover, self-compassion has been shown to reduce state anxiety in various social contexts (Harwood and Kocovski, 2017) and functions as a resource for emotion regulation, helping individuals reappraise stressful situations and reduce negative emotions (Luo et al., 2019). Moreover, research on university students has shown that enhancing self-compassion among this group can effectively increase psychological resilience in the face of stress and reduce anxiety levels (DiFonte et al., 2024). Therefore, we believe that self-compassion may help alleviate English learning anxiety among university student.

Personal growth initiative refers to an individual's intentional and active involvement in self-change and development, encompassing four core dimensions: readiness for change, planfulness, resource utilization, and intentional behavior (Robitschek, 1998). Previous studies have shown that self-compassion significantly enhances college students' self-efficacy (Smeets et al., 2014), which in turn is a vital psychological resource for promoting proactive growth behaviors. Indeed, research has found a significant positive correlation between self-efficacy and personal growth initiative (Kyriazos and Poga, 2024), and low self-efficacy may lead to anxiety during English speaking practice (Pang, 2020). This suggests that self-compassion may foster personal growth initiative by enhancing learners' self-efficacy. Moreover, self-determination theory posits that individuals possess an inherent tendency toward growth and development, which is realized through the satisfaction of three basic psychological needs: autonomy, competence, and relatedness (Ryan and Deci, 2000). This theory suggests that when learners' basic needs are met, they are more likely to experience intrinsic motivation, effective emotion regulation, and reduced anxiety (Alamer and Almulhim, 2021). Research has confirmed that when learners' needs are supported, they are more likely to develop effective coping strategies, enhance personal growth initiative, and strengthen learning autonomy (Shelton-Strong, 2025). Thus, self-compassion facilitates the fulfillment of basic psychological needs and aligns closely with the growth-oriented dynamics proposed in self-determination theory. Consequently, individuals with higher self-compassion are more likely to experience need satisfaction, which in turn fuels intrinsic motivation and strengthens personal growth initiative. Taken together, it can be inferred that individuals with higher personal growth initiative are better equipped to cope with academic stress and challenges, thereby reducing English learning anxiety.

Experiential avoidance, a construct introduced by Hayes, refers to individuals' attempts to evade, suppress, control, or eliminate unpleasant internal experiences such as emotions, thoughts, memories, or sensations through both internal and external strategies [20]. Studies suggest that experiential avoidance is a maladaptive form of emotion regulation, often associated with higher levels of negative emotions, stress, and psychological distress (Fergus et al., 2013). This may lead to students with higher levels of experiential avoidance being more likely to experience anxiety in the context of language learning. According to emotion regulation theory, regulatory strategies can be categorized into situation selection, situation modification, attentional deployment, cognitive change, and response modulation (Gross James, 1998). These factors influence emotional experiences in language learning. For example, adaptive strategies (such as acceptance and reappraisal) can enhance coping ability, while maladaptive strategies (such as avoidance and suppression) can exacerbate anxiety (Tamir and Mauss, 2010; Wolgast et al., 2013; Zhang et al., 2025). Among these strategies, cognitive change and response modulation are considered central pathways for achieving adaptive outcomes. Previous research has demonstrated that self-compassion improves cognitive appraisal and regulatory skills, thereby reducing maladaptive behavioral avoidance and encouraging more adaptive emotion regulation strategies (Paucsik et al., 2023). Therefore, we believe that individuals with higher levels of self-compassion are more likely to adopt adaptive and constructive emotion regulation strategies, reducing their tendency toward experiential avoidance. This helps individuals cope more effectively with external stress and anxiety-inducing situations, ultimately lowering their English learning anxiety.

Evidence suggests that personal growth initiative enables individuals to adopt more adaptive cognitive patterns in adverse and challenging circumstances, fostering a sense of control and confidence when coping with or altering unfavorable contexts (Blackie et al., 2015). In contrast, individuals who are unwilling to actively grow often struggle to overcome setbacks and learn from criticism, making them more prone to avoidance behaviors and experiencing poorer mental health (Sung et al., 2017). Besides, according to self-determination theory, the emotional support and self-acceptance provided by self-compassion help fulfill individuals‘ psychological needs. When these basic psychological needs are satisfied, adaptive behaviors are promoted, thereby enhancing motivation for growth (Nalipay et al., 2020; Namaziandost et al., 2024). This process allows individuals to approach challenges more positively, reduce anxiety and stress. Research has also shown that proactive personal growth is positively correlated with students' positive coping strategies and self-efficacy (Weigold et al., 2024). Individuals with higher levels of personal growth initiative tend to adopt emotional regulation strategies that enhance enjoyment in foreign language learning, reduce anxiety, and stimulate higher motivation to learn (Alqarni, 2024). These individuals demonstrate stronger coping abilities when facing difficulties, preferring to take proactive steps rather than avoid problems. In summary, we believe that individuals with higher personal growth initiative tend to have lower levels of experiential avoidance. Therefore, in our study, rather than a parallel mediation, we propose that personal growth initiative and experiential avoidance may play a chained mediating role between self-compassion and English learning anxiety.

Gender, as a key sociocultural variable, has long been recognized as influencing emotion regulation, stress responses, and language learning. Previous studies have reported that females tend to exhibit higher levels of anxiety than males when learning a foreign language, which can hinder their language learning and overall performance (Dewaele and Macintyre, 2014; Park and French, 2013; Rababah and Almwajeh, 2024). This anxiety may be attributed to their heightened emotional sensitivity and higher expectations regarding language achievement. Furthermore, research suggests that women are more likely to employ emotion-focused coping strategies, such as avoidance or self-blame, whereas men tend to adopt problem-focused strategies (Matud, 2004; Tamres et al., 2002). Regarding avoidance tendencies, women often display higher levels of experiential avoidance, which makes them more vulnerable to anxiety in high-pressure contexts (Cooper et al., 2022). A study in China also found that, compared to men, women tend to use fewer adaptive emotion regulation strategies to cope with anxiety (Yao et al., 2024). Considering well-established gender differences in emotion regulation (Yao et al., 2024), it is plausible that gender moderates the relationship between experiential avoidance and English learning anxiety. Specifically, in the face of challenges and uncertainties in language learning, experiential avoidance may exert a stronger predictive effect on anxiety among women than among men.

**Figure 1:**
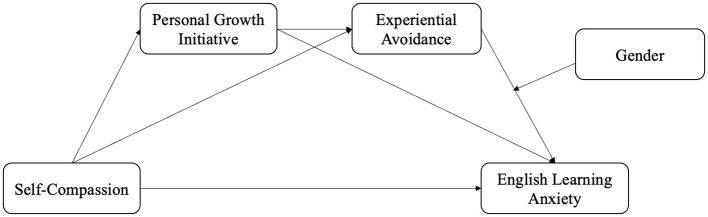
Proposed model.

In summary, based on the existing literature, this study constructs a chained mediation model in which self-compassion predicts English learning anxiety through personal growth initiative and experiential avoidance as mediators, with gender included as a moderator (Figure 1). The proposed model is grounded in a synthesis of self-determination theory and emotion regulation theory, which offer complementary perspectives on the psychological mechanisms underlying English learning anxiety among Chinese university students. Drawing from self-determination theory, we hypothesize that self-compassion acts as an antecedent psychological resource that enhances personal growth initiative by fulfilling students' basic psychological needs for autonomy, competence, and relatedness. This, in turn, is expected to translate motivational support into proactive, goal-oriented behaviors, which foster adaptive emotion regulation and reduce experiential avoidance. According to emotion regulation theory, we also suggest that experiential avoidance—viewed as a maladaptive form of emotion regulation—represents a downstream outcome in this sequence, potentially exacerbating anxiety when not managed effectively. By integrating these constructs, we aim to clarify how personal growth initiative functions as a bridge between the motivational processes initiated by self-compassion and the regulatory tendencies associated with experiential avoidance, ultimately contributing to a reduction in English learning anxiety. This unified framework thus provides a rationale for why self-compassion may not only enhance motivation but also improve emotional regulation strategies, potentially offering a pathway to alleviate learning anxiety. Therefore, based on the literature and the theoretical model, we aim to further test and validate these relationships through empirical analysis. The hypotheses are as follows:

Hypothesis 1: Self-compassion significantly and negatively predicts English learning anxiety.

Hypothesis 2: Personal growth initiative mediates the relationship between self-compassion and English learning anxiety.

Hypothesis 3: Experiential avoidance mediates the relationship between self-compassion and English learning anxiety.

Hypothesis 4: Personal growth initiative and experiential avoidance constitute a chained mediation pathway between self-compassion and English learning anxiety, such that self-compassion enhances personal growth initiative, which in turn reduces experiential avoidance, thereby lowering English learning anxiety.

Hypothesis 5: Gender moderates the relationship between experiential avoidance and English learning anxiety, such that the predictive effect of experiential avoidance on anxiety varies across genders.

## Method

### Participants

This study was approved by the university's ethics committee. A convenience sampling method was used to recruit participants from universities across 26 provinces and municipalities in China. The inclusion criteria for participants were as follows: (1) university students currently enrolled in undergraduate or graduate programs, (2) aged 18 years and above, (3) who have studied English as part of their academic curriculum, and (4) willing to participate in the study by completing the survey. A total of 1,585 questionnaires were distributed. After excluding invalid responses (e.g., careless answering, incomplete responses, patterned responses), 1,287 valid questionnaires were retained, resulting in an effective response rate of 81.20%. Of the participants, 616 were male (47.90%) and 671 were female (52.10%), with ages ranging from 18 to 30 years (*M* = 20.44, SD = 1.72). Among the participants, 656 students (51.00%) were from urban areas, 383 students (29.80%) were from rural areas, and 248 students (19.30%) were from townships.

## Measures

### Self-Compassion Scale

The Self-Compassion Scale developed by Neff K. D. (2003) and revised by Chen et al. (2011) was used to measure individuals' understanding and acceptance of their own suffering. The scale consists of 12 items, such as “I have a critical attitude toward my flaws and shortcomings” and “When painful events occur, I try to view them objectively.” A 5-point Likert scale was used, ranging from 1 (“Strongly disagree”) to 5 (“Strongly agree.”) Higher total scores indicate greater ability to self-soothe and show self-kindness in response to negative emotional experiences. Items 1, 2, 3, 4, 6, 8, 11, 13, 16, 18, 20, 21, 24, 25 are reverse-scored. The Cronbach's alpha coefficient for this scale in the present study was 0.85.

### Personal Growth Initiative Scale

The Personal Growth Initiative Scale, developed by Robitschek (1998) and revised by Tian (2016), was employed to assess personal growth initiative. The scale includes 16 items covering four dimensions: planning, readiness for change, resource utilization, and proactive behavior (e.g., “I strive to continuously improve myself”). The scale uses a 6-point scoring system, ranging from 0 (“Strongly disagree”) to 5 (“Strongly agree”). Higher total scores reflect greater personal growth initiative. The Cronbach's alpha coefficient for this scale in the present study was 0.94.

### Experiential Avoidance Scale

The Acceptance and Action Questionnaire-II (AAQ-II) (Cao et al., 2013) was used to measure experiential avoidance. The scale consists of 7 items, rated on a 7-point scale from 1 (“Never”) to 7 (“Always”). The total score ranges from 7 to 49, with higher scores indicating greater experiential avoidance and lower psychological flexibility. The Cronbach's alpha coefficient for this scale in the present study was 0.91.

### English Learning Anxiety Scale

The Foreign Language Classroom Anxiety Scale developed by Horwitz et al. (1986) and revised by Da (2007) was used to assess English learning anxiety. This scale contains 23 items, with four subscales: nervousness, worry, fear of asking questions, and general anxiety. For example, “I feel afraid when I don't understand what the teacher says in English.” A 5-point Likert scale was used, ranging from 1 (“Strongly disagree”) to 5 (“Strongly agree”). Higher total scores indicate greater English learning anxiety. The Cronbach's alpha coefficient for this scale in the present study was 0.95.

### Data analysis

Data were analyzed using SPSS 26.0 for descriptive statistics, difference testing, and correlation analysis. The moderated and mediating effects were tested using the Process macro version 3.5. Because the data were collected through self-report methods, common method bias was tested using Harman's single-factor test (Podsakoff et al., 2003). Without rotation, 10 common factors with eigenvalues greater than 1 were extracted. The first common factor explained 27.20% of the variance, which is far below the 40% threshold, indicating that no significant common method bias existed in this study. Parameter estimation was carried out using the bootstrap method with 5,000 bootstrap samples, providing a 95% confidence interval (CI). An effect was considered significant if the CI did not contain zero, indicating a statistically reliable effect, and not significant if the CI included zero, suggesting no significant effect. A chain mediation model was tested using the Process macro Model 6 (Hayes, 2017), with age, family location, and parental education level included as control variables. A moderated chain mediation model was tested using Process macro Model 87 (Hayes, 2017), with age, family location, and parental education level included as control variables.

## Results

### Descriptive statistics and correlations

Table 1 presents the descriptive statistics and correlation analysis results for the study variables. Self-compassion was significantly positively correlated with personal growth initiative and negatively correlated with experiential avoidance and English learning anxiety. Personal growth initiative showed a significant negative correlation with both experiential avoidance and English learning anxiety, while experiential avoidance was significantly positively correlated with English learning anxiety.

**Table 1:** Descriptive statistics and correlation analysis of variables.

**Variable**	***M***	***SD***	**1**	**2**	**3**
1 Self-compassion	85.16	10.86	-		
2 Personal growth initiative	72.60	10.26	0.51^***^	-	
3 Experiential avoidance	28.41	8.63	−0.66^***^	−0.41^***^	-
4 English learning anxiety	66.41	18.03	−0.45^***^	−0.34^***^	0.59^***^

*N* = 1287, ^***^p < 0.001.

Results of One-Way ANOVA showed that significant differences were found in self-compassion (*F*_(2, 1284)_ = 7.64, *p* < 0.001), personal growth initiative (*F*_(2, 1284)_ = 9.84, *p* < 0.001), experiential avoidance (*F*_(2, 1284)_ = 18.91, *p* < 0.001), and English learning anxiety (*F*_(2, 1284)_ = 17.23, *p* < 0.001) across students from different areas (urban, rural, and township). Additionally, significant differences in self-compassion (*F*_(11, 1275)_ = 5.40, *p* < 0.001) and personal growth initiative (F _(11, 1275)_ = 11.61, *p* < 0.001) were found across different age groups. The education level of fathers significantly influenced self-compassion (*F*_(5, 1281)_ = 9.75, *p* < 0.001), personal growth initiative (*F*_(5, 1281)_ = 20.20, *p* < 0.001), experiential avoidance (*F*_(5, 1281)_ = 7.97, *p* < 0.001), and English learning anxiety (*F*_(5, 1281)_ = 10.29, *p* < 0.001). Similarly, the education level of mothers significantly influenced self-compassion (*F*_(5, 1281)_ = 6.17, *p* < 0.001), personal growth initiative (*F*_(5, 1281)_ = 17.03, *p* < 0.001), experiential avoidance (*F*_(5, 1281)_ = 4.95, *p* < 0.001), and English learning anxiety (*F*_(5, 1281)_ = 7.09, *p* < 0.001). Given the impact of these variables on the study variables, they were included as control variables in the model.

### Testing the chain mediation model

The results (see Table 2) indicated a significant negative relationship between self-compassion and English learning anxiety (β = −0.43, *p* < 0.001). When personal growth initiative and experiential avoidance were included in the model, self-compassion still significantly predicted English learning anxiety negatively (β = −0.07, *p* = 0.022). Additionally, self-compassion significantly predicted personal growth initiative (β = 0.47, *p* < 0.001) and experiential avoidance (β = −0.60, *p* < 0.001); personal growth initiative significantly predicted experiential avoidance (β = −0.11, *p* < 0.001) and English learning anxiety (β = −0.09, *p* < 0.001); and experiential avoidance significantly predicted English learning anxiety (β = 0.49, *p* < 0.001). Mediation analysis showed that personal growth initiative mediated the effect of self-compassion on English learning anxiety (indirect effect = −0.044, accounting for 10.14% of the total effect), while experiential avoidance mediated this effect (indirect effect = −0.293, accounting for 67.51% of the total effect). Furthermore, the chained mediation effect of personal growth initiative and experiential avoidance was significant (indirect effect = −0.024, accounting for 5.53% of the total effect) (see Table 3).

**Table 2:** Chain mediation model test of personal growth initiative and experiential avoidance.

**Regression equation**		**Overall fit indices**			**Significance of the regression coefficients**	
**Outcome variables**	**Predictors**	***R***	***R** ^2^*	***F***	β	***95%CI***
English learning anxiety	Self-compassion	0.47	0.22	72.49^***^	−0.43	−17.28^***^
Personal growth initiative	Self-compassion	0.56	0.32	119.82^***^	0.47	19.78^***^
Experiential avoidance	Self-compassion	0.67	0.45	172.71^***^	−0.60	−24.88^***^
	Personal growth initiative				−0.11	−4.26^***^
English learning anxiety	Self-compassion	0.61	0.37	105.70^***^	−0.07	−2.30^*^
	Personal growth initiative				−0.09	−3.48^***^
	Experiential avoidance				0.49	16.28^***^

*N* = 1287,^*^p < 0.05,^**^p < 0.01, ^***^p < 0.001. Age, home location, mother and father education level were done as control variables included in the model. The study variables were standardized.

**Table 3:** Chain mediation model effect size analysis.

**Effect type**	***Effect***	***SE***	***95%CI***
Total effect	−0.434	0.03	[-0.484,−0.385]
Direct effect	−0.073	0.03	[-0.135,−0.011]
Indirect effect of personal growth initiative experiential avoidance	−0.044	0.01	[-0.072,−0.016]
Indirect effect of experiential avoidance	−0.293	0.02	[-0.338,−0.251]
Indirect effect of personal growth initiative and experiential avoidance	0.024	0.01	[-0.037,−0.012]

Effect size. SE and 95% CI refer to the standard errors, lower and upper 95% confidence intervals of the indirect effects estimated by the bias-corrected percentile Bootstrap method, respectively.

### Testing the moderated chain mediation model

The results (see Table 4) showed that the interaction between experiential avoidance and gender significantly predicted English learning anxiety (β = 0.11, *p* = 0.014).

**Table 4:** Moderated chain mediation model test.

**Regression equation**		**Overall fit indices**			**Significance of the regression coefficients**	
**Outcome variables**	**Predictors**	***R***	***R** ^2^*	***F***	β	***95%CI***
Personal growth initiative	Self-compassion	0.56	0.32	119.82^***^	0.47	19.78^***^
Experiential avoidance	Self-compassion	0.67	0.45	172.71^***^	−0.60	−24.88^***^
	Personal growth initiative				−0.11	−4.26^***^
English learning anxiety	Self-compassion	0.62	0.38	87.47^***^	−0.08	−2.61^**^
	Personal growth initiative				−0.10	−3.86^***^
	Experiential avoidance				0.31	4.00^***^
	gender				0.22	4.79^***^
	Experiential Avoidance gender				0.11	2.45^*^

*N* = 1287,^*^p < 0.05,^**^p < 0.01,^***^p < 0.001. Age, home location, mother and father education level were done as control variables included in the model. The study variables were standardized.

### Simple slope analysis

**Figure 2:**
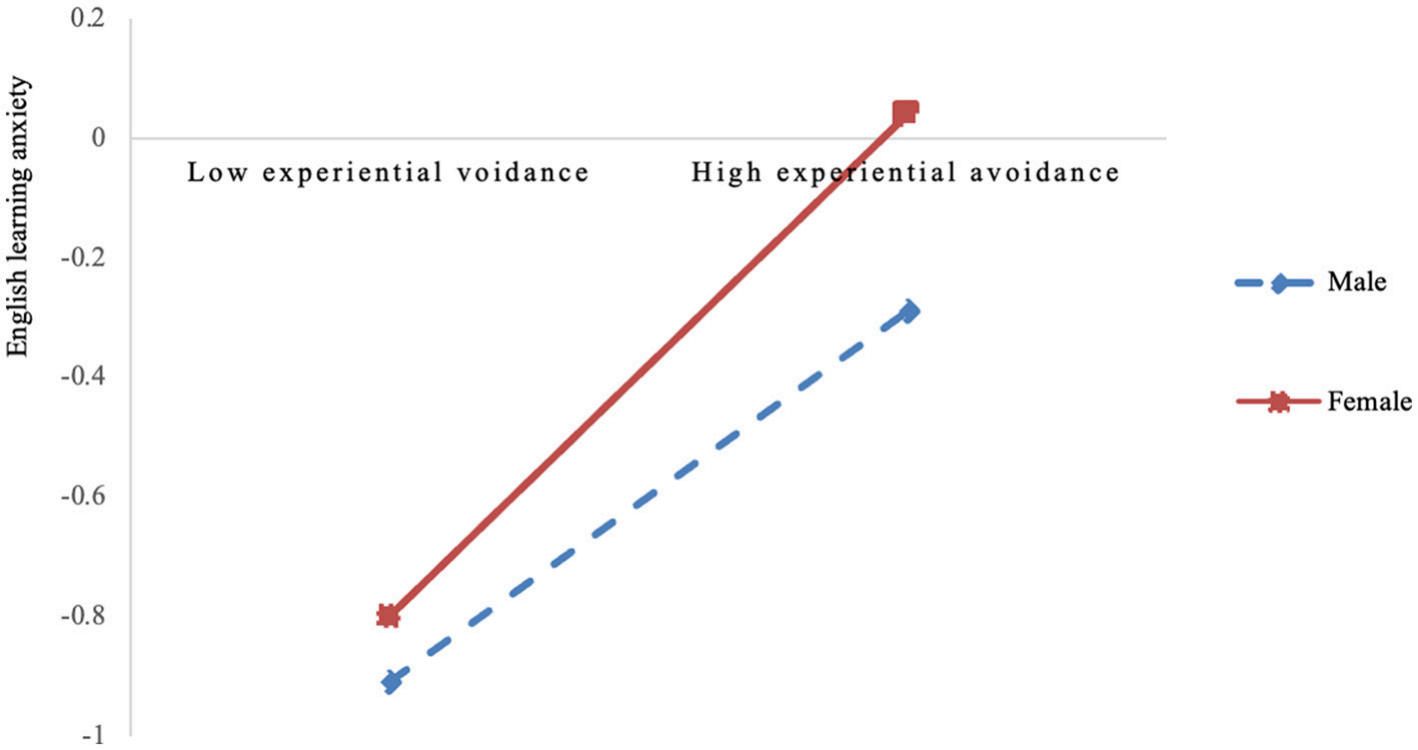
The moderating role of gender between experiential avoidance and English learning anxiety.

To further explain the interaction between experiential avoidance and gender on English learning anxiety, simple slope analysis was conducted (see Figure 2). The results indicated that for male students, experiential avoidance significantly predicted English learning anxiety (simple slope = 0.42, *p* < 0.001); for female students, experiential avoidance had a stronger predictive effect on English learning anxiety (simple slope = 0.53, *p* < 0.001).

## Discussion

This study explored the impact of self-compassion on English learning anxiety and its underlying mechanisms. The results indicate that self-compassion significantly and negatively predicts English learning anxiety, with this effect mediated by personal growth initiative and experiential avoidance. Specifically, higher levels of self-compassion promoted an increase in personal growth initiative and effectively reduced experiential avoidance, thereby alleviating anxiety during English learning. Further analysis revealed that experiential avoidance played a key mediating role between self-compassion and learning anxiety. Gender also moderated this process, particularly for female students, who were more likely to amplify their anxiety through avoidance behaviors. Thus, this study not only deepens the understanding of the relationship between self-compassion and English learning anxiety but also highlights the roles of personal growth initiative and gender differences in this context, providing theoretical support for future strategies to address English learning anxiety.

The results of this study show that self-compassion significantly negatively predicts English learning anxiety. This finding supports the efficacy of self-compassion in alleviating anxiety and stress. Meta-analytic studies have demonstrated that self-compassion has a small to moderate effect on reducing anxiety and stress, with continued alleviating effects even at follow-up assessments (Han and Kim, 2023). English learning anxiety, as a complex emotional reaction triggered by specific classroom situations, is primarily composed of three core components: communication apprehension, test anxiety, and fear of negative evaluation (Horwitz et al., 1986). This finding is consistent with research showing that self-compassion plays a significant role in alleviating anxiety in various contexts, particularly in language learning, where it can significantly reduce anxiety induced by situational pressure (Harwood and Kocovski, 2017; Long and Neff, 2018).

Additionally, personal growth initiative mediated the relationship between self-compassion and English learning anxiety. Previous studies have shown that self-compassion not only enhances personal growth initiative but also significantly reduces anxiety (Dundas et al., 2017). This finding aligns with the positive effects of self-compassion on individual psychological development. Moreover, personal growth initiative is associated with lower levels of social anxiety and negative emotions, higher levels of positive emotions, and reduced self-differentiation (Hardin et al., 2007). When individuals reduce social anxiety, they are more likely to engage in communication during English learning, and their lower negative emotions and higher positive emotions provide continuous positive feedback, helping them handle learning challenges more effectively. More importantly, personal growth initiative is positively related to autonomy (Cankaya et al., 2018). According to self-determination theory, when individuals perceive their autonomy is supported, they are more likely to exhibit positive learning motivation and emotion regulation. Enhanced autonomy in English learning helps students maintain lower anxiety levels and face challenges with greater confidence (Üztemur, 2020). Therefore, through self-compassion, individuals experience support that fosters personal growth initiative, which helps them overcome difficulties during English learning and reduce anxiety.

This study also confirmed that experiential avoidance mediates the relationship between self-compassion and English learning anxiety. One of the core elements of self-compassion is mindfulness, and mindfulness-based self-compassion interventions have been shown to effectively increase self-compassion, reduce experiential avoidance, and alleviate anxiety and depression, while enhancing positive emotions (Yela et al., 2022). Thus, by enhancing self-compassion, individuals are better able to view sources of stress and emotional responses rationally, reducing avoidance behaviors, especially in the context of English learning, where students can better cope with the anxiety caused by language barriers and social interactions. Furthermore, self-compassion is related to emotion regulation capacity and psychological flexibility, which helps to enhance psychological resilience and reduce internalized shame, thereby reducing experiential avoidance behaviors (Cuerda-Ballester et al., 2024; Williams et al., 2021). Studies have confirmed that experiential avoidance is linked to several psychological symptoms (Akbari et al., 2022). By avoiding negative emotions and sources of stress, individuals temporarily alleviate discomfort, but this avoidance behavior exacerbates psychological distress, leading to long-term emotional problems (Plumb et al., 2004). It also results in higher test anxiety, further leading to avoidance behaviors and obstructing English learning (Hilz et al., 2025). This vicious cycle not only hinders the development of language skills but also prevents students from employing coping strategies to handle learning difficulties. Students with higher self-compassion, however, tend to have more adaptive emotion regulation strategies (Paucsik et al., 2023), and reduced levels of experiential avoidance, which contributes positively to alleviating English learning anxiety.

Furthermore, this study verified that personal growth initiative and experiential avoidance form a chained mediation pathway between self-compassion and English learning anxiety. Specifically, self-compassion enhances personal growth initiative, which in turn reduces experiential avoidance, leading to reduced English learning anxiety. This result resonates with the work of Dweck, who noted that fixed mindset reinforces experiential avoidance, thereby affecting psychological wellbeing (Sung et al., 2017). In contrast, this study demonstrates that by enhancing personal growth initiative through self-compassion, not only does avoidance behavior decrease, but individual coping strategies also improve, thereby reducing anxiety levels. This difference reflects the unique role of self-compassion in promoting positive responses to learning challenges and reducing emotional avoidance. Unlike the traditional exam-oriented learning model, personal growth initiative emphasizes self-driven learning and the cultivation of psychological adaptation. Studies have shown that personal growth initiative enhances students' self-efficacy and their ability to cope with challenges (Weigold et al., 2024, 2020). When students feel they can actively control their learning process and achieve growth through proactive efforts, their anxiety levels significantly decrease. This explains why enhancing personal growth initiative reduces experiential avoidance behaviors—there is less tendency to avoid emotions and tasks, and students begin to face English learning challenges more positively. Moreover, experiential avoidance, as a typical emotion regulation strategy, makes individuals more vulnerable under pressure if relied upon long-term, leading them to fall into anxiety and self-doubt. By improving self-compassion and personal growth initiative, students not only learn more adaptive emotion regulation strategies but also cultivate greater psychological resilience, enabling them to face the uncertainties and challenges of English learning with confidence.

Additionally, this study found that the predictive effect of experiential avoidance on English learning anxiety varies between different gender groups. Specifically, experiential avoidance had a stronger predictive effect on English learning anxiety in female students. This phenomenon may be attributed to multiple factors. On one hand, in traditional Chinese culture, women often face greater family responsibilities and societal role pressures, especially in terms of self-expression and academic achievement. Studies have shown that women generally have lower levels of self-confidence compared to men, and this internal self-doubt and lack of confidence may lead to higher anxiety when facing challenges in foreign language learning (Xu et al., 2025). Furthermore, from a neurobiological perspective, men and women respond differently to stress physiologically. Research has shown that the amygdala in women's brains (which is associated with emotional responses and anxiety) is typically more active, while the prefrontal cortex (responsible for emotional regulation and rational judgment) in men tends to be more active (Kinrys and Wygant, 2005; Kuehner, 2017). This difference suggests that women may exhibit stronger emotional reactions when facing external pressures, such as anxiety in language learning. In the context of foreign language learning, these factors may lead to women experiencing higher levels of anxiety compared to men. Moreover, under academic and social pressures, women are more likely to show emotional reactions and anxiety, with a stronger need for external support and understanding. However, as society is more tolerant of emotional expression from women, they may be more likely to exhibit such avoidance behaviors, particularly when facing learning pressure. In contrast, due to social norms, men tend to express less anxiety, as society expects them to present an image of resilience and invulnerability. This cultural pressure leads men to be less willing or less able to express anxiety (Fisher et al., 2022). This shaping of gender roles may explain why men report less English learning anxiety, even if they face similar psychological pressures.

In conclusion, this study not only theoretically extends the understanding of the relationship between self-compassion and English learning anxiety but also provides practical intervention suggestions in educational contexts. From a theoretical perspective, this study constructed a chained mediation model based on self-compassion, revealing how self-compassion reduces English learning anxiety through the enhancement of personal growth initiative and reduction of experiential avoidance. This research not only enriches the application of self-compassion theory in foreign language learning but also provides a new perspective on the role of gender in this process. Additionally, it highlights the central role of personal growth initiative in alleviating learning anxiety and advances the integration of self-compassion and personal growth theories, offering new directions for future research.

From a practical perspective, this study provides educators with effective emotion regulation strategies, especially within the Chinese exam-oriented educational context, where students face high levels of learning pressure and anxiety. The findings suggest that self-compassion can significantly alleviate anxiety during English learning. Therefore, educators can introduce self-compassion concepts into classroom teaching through mindfulness meditation and emotion regulation exercises, helping students better cope with anxiety (Egan et al., 2022). Moreover, teachers should consider gender differences and design personalized psychological interventions. For female students, more emotional support can be provided to encourage them to face challenges actively in their studies, while for male students, efforts should be made to promote healthy emotional expression and effective emotion management.

Despite its contributions, this study has some limitations.

First, this study was not pre-registered, which may affect the transparency and reproducibility of the analytic process. Future studies could benefit from pre-registering their hypotheses and analysis plans to enhance the rigor and reproducibility of the findings. Second, the sample was limited to Chinese university students, and the convenience sampling method used may limit the external validity of the findings. Future research could expand the sample to include students from various regions and cultural backgrounds to enhance the generalizability of the results. Third, while self-report questionnaires were used to gather data, which allows for direct insight into students' psychological states and learning conditions, they may also be susceptible to social desirability bias and self-reporting bias. Future research could consider using a combination of data collection methods, such as physiological measures and behavioral observation, to improve the objectivity of the findings. Moreover, this study employed a cross-sectional design, which does not establish causal relationships, so it is not possible to fully verify whether self-compassion directly causes a reduction in English learning anxiety. Longitudinal or experimental designs could further verify the long-term effects of self-compassion on English learning anxiety and its causal pathways.

## Conclusion

This study thoroughly explored the relationships between self-compassion, personal growth initiative, experiential avoidance, and English learning anxiety, and verified the indirect impact of self-compassion on English learning anxiety through personal growth initiative and experiential avoidance. The results indicate that self-compassion significantly negatively predicts English learning anxiety, with personal growth initiative and experiential avoidance playing crucial mediating roles in this relationship. Furthermore, gender differences moderated the relationship between experiential avoidance and English learning anxiety, with experiential avoidance having a stronger predictive effect on English learning anxiety for female students. Future research can not only improve in terms of data collection and research design, but also further explore intervention studies based on core variables such as self-compassion, personal growth initiative, and experiential avoidance. These studies can not only verify the effectiveness of these variables in reducing English learning anxiety, but also provide guidance for teaching practices.

## Data Availability

The raw data supporting the conclusions of this article will be made available by the authors, without undue reservation.
